# Undergraduate dental implantology education in the United Kingdom – looking to the past to plan for the future

**DOI:** 10.1038/s41415-026-9627-5

**Published:** 2026-05-08

**Authors:** Elizabeth King, Katarzyna Gurzawska-Comis, Gerry Mckenna, Charlotte Stilwell

**Affiliations:** 466314056585827373535 University of Bristol, Bristol, United Kingdom; 783436979883713540933 Department of Dentistry and Oral Health, Aarhus University, Aarhus, Denmark; Department of Musculoskeletal and Ageing Science and School of Dentistry, Liverpool Head and Neck Centre, University of Liverpool, Liverpool, United Kingdom; 597130158509457211060 Queens University Belfast, Belfast, United Kingdom; 067999546743674409808 University of Geneva, Geneva, Switzerland

## Abstract

Dental implantology has traditionally been a postgraduate subject; however, as dental implants become an increasingly popular tooth replacement option for dentists and patients, there is a growing need for newly graduated dentists to be familiar with core aspects of implant dentistry. Furthermore, as the field of implant dentistry advances, so has the breadth of treatment indications and complexity. Therefore, not only are there more implant patients, there are more complex implant patients in the community. The care of implants and treatment of peri-implant tissues falls within the scope of the general dental practitioner (GDP), and it is widely considered that GDPs have a duty of care to monitor and maintain dental implants. It is important that undergraduate dental education reflects these evolutions in clinical dentistry to equip newly graduated dentists with the competencies to identify, monitor and maintain dental implants. In recognition of the continuing challenges concerning the delivery of undergraduate dental implant education in the UK and Ireland, this article aims to reflect on progress over the past 40 years, examine its status today and identify the future education priorities to be addressed.

**Supplementary Information:**

Zusatzmaterial online: Zu diesem Beitrag sind unter 10.1038/s41415-026-9627-5 für autorisierte Leser zusätzliche Dateien abrufbar.

## Key points

- Significant improvements have been achieved for undergraduate education in the UK and Ireland over the past 40 years, with implant dentistry embedded in the curriculum in all schools.
- There continues to be a wide variation in teaching methodologies, teaching hours and clinical teaching within the UK and Ireland, both between schools and in comparison to Europe and the wider world.
- Continued curricula guidance on dental implantology is essential, including guidance on learning styles, format, environment and appropriate assessment to encourage schools to make effective curriculum changes.

**Supplementary Information:**

Zusatzmaterial online: Zu diesem Beitrag sind unter 10.1038/s41415-026-9627-5 für autorisierte Leser zusätzliche Dateien abrufbar.

## Introduction

Developing dental implantology as a core topic for UK dental undergraduate (UG) education has been a topic of debate for a number of years. As set out in the General Dental Council (GDC) *Preparing for Practice* document, dental UG students should be taught a ‘range of professional skills required to begin working as part of a dental team and be well prepared for independent practice'.^1^ From September 2025, *Preparing for Practice* was superseded by the *Safe Practitioner Framework*. The GDC states that the learning outcomes in the safe practitioner ‘have been designed to allow education providers flexibility to use their expertise to develop programme curricula and to amend these to reflect changes in practice over time'. Furthermore, they state, ‘the role of the education provider is to determine the right areas to teach and assess within the remit of that professional group at the level expected to be a safe practitioner'. Therefore, to ensure newly qualified dentists are properly equipped to safely manage patients, it is important that their UG education reflects evolutions in clinical dentistry.

Dental implantology has traditionally been considered a postgraduate (PG) subject. As the population of patients with dental implants is continuously and rapidly increasing, however, there is emerging need for the wider dental team to understand principles of dental implantology treatment. The 2009 Adult Dental Health Survey recorded dental implants for the first time and the results showed that 1% of the UK population had at least one dental implant.^2^ In 2012, the Association of Dental Implantology estimated that as many as 130,000 individual implants were being placed every year.^3^ The 2021 Adult Oral Health Survey commissioned by Public Health England (now the Office for Health Improvement and Disparities), showed that 5% of adults reported that they had a dental implant.^4^ Correlating this percentage to the population of England in 2021 (56.5 million) estimates that around 2,825,000 individuals in England had a dental implant in 2021. As the number of dental implants in the patient population has increased, so too has the scope of dental implant treatment with regards to case selection and complexity. Therefore, not only will more implants present in general dental practice, the complexity and diversity of implant cases will also increase.

In recognition of the continuing challenges concerning the delivery of UG implant education in the UK and Ireland, this article aims to reflect on progress over the past 40 years, examine its status today and identify the future priorities to be addressed.

### The role and responsibility of the general dental practitioner

As dental implants become more accessible to patients, there is an increasing need for general dental practitioners (GDPs) to understand the core aspects of dental implant treatment and maintenance. The 2013 GDC scope of practice, which sets out the skills and abilities of each dental professional, identifies that:

- Dentists should undertake ‘care of implants and treatment of peri-implant tissues'
- Dentists with additional skills can provide implants.

There is, therefore, a professional and public expectation that general dentists have a responsibility to monitor and maintain dental implants in the long-term.

The GDC *Safe Practitioner Framework* specifies that a graduating UK dentist should be able to ‘explain the use of implants as a treatment option, including their outcomes, limitations, and risks'. Similarly, a graduating dental therapist should be able to ‘describe conditions or complications that may arise following dental implant therapy' and ‘manage the health of peri-implant tissues'.^5^ Consequently, GDPs are expected to understand the indications and treatment planning for dental implants (including surgical protocol and restoration) in order to provide appropriate patient advice, consent and referral, and dental therapists should be competent at assessing and maintaining the peri-implant tissues.

In a recent publication discussing GDPs' duty of care for the ongoing success or failure of any pre-existing implants, it was acknowledged that GDPs ‘will have a duty of care to, at the very least, monitor these implants', particularly if the dentist who placed the implant/s has not organised ongoing maintenance.^6^ A similar subject was discussed by the European Association of Osseointegration, whereby the education of professionals and patients to improve diagnosis and prevention of preimplant disease was considered high priority in recent guidelines for development of implant dentistry in the next ten years.^7^

With the expectation that GDPs can play a central and effective role in managing patients with dental implants comes the expectation that they possess the corresponding knowledge and skills required at the point of graduation to be able to practice safely from the outset. This clearly poses an education and training requirement to be accommodated within the respective bachelor's degrees in dental surgery and dental therapy.

### A brief history of undergraduate education in the UK and Ireland

**Fig. 1:**
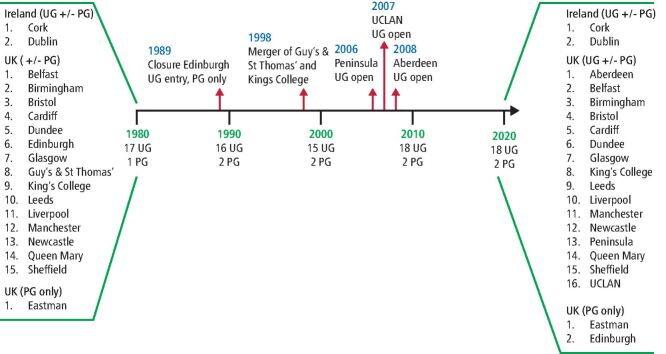
Changes in the number of UK dental schools from 1980 to present

Implant teaching has existed to some degree within UG education in the UK since 1985.^8^ Numerous surveys have been conducted in the intervening four decades since then to understand the scope and evolution of UG implant education in the UK and Ireland. Online Supplementary Table 1 summarises the results from each survey including methods of learning, topics covered and time allocated for implant teaching. Over the time period in which the surveys were conducted, there have been changes in the number of dental schools in the UK due to mergers and new openings (see Fig. 1).

In a survey from in 1993, Watson assessed teaching of implant dentistry in the then 18 schools of the UK and Ireland.^8^ Of the 17 responding schools, 12 (70%) reported undertaking UG implant teaching which was shared between the restorative and oral surgery teams. All schools which taught implant dentistry used lectures, whereas clinical education was limited to observation in fewer than half the schools (six schools − 35%). The opportunity for observation of implant surgery was only provided for some students in two schools.

Young *et al.* (1999) conducted a similar survey, evaluating PG and UG training in oral implantology in the UK and Ireland.^9^ The authors contacted 16 (of the 18 dental schools in the UK or Ireland at the time of the survey) for responses but did not specify which country they were based in. All 16 contacted schools (100%) responded that they had some level of UG teaching in implant dentistry. Compared to the 1993 survey, however, clinical implant education had reduced with only four schools (25%) providing some degree of observation. The authors emphasised a variation in the survey findings regarding course content and clinical opportunities for students both within and between schools.

In 2008, Addy *et al.* surveyed 15 dental schools (of the then total of 17 dental schools at the time of the survey) within UK and Ireland to evaluate implant teaching.^10^ Again, there were no details of which dental schools had been surveyed. All 15 dental schools responded, with 13 (87%) reporting that they provided some level of teaching in implant dentistry. This was a decrease from the 100% reported by the Young *et al.* survey in 1999.^9^ The variety of teaching methods, on the other hand, had increased, with lectures (60%), simulation on phantom heads (53%) and clinical teaching (46%) being provided in some schools. Clinical education also included a wider range of activities, with treatment planning (46%), restoration (46%) and surgery (33%) taught in some school. Of the schools providing clinical education, most offered opportunities for observation (restoration 46%; surgery 33%) while in four schools (27%) UGs performed implant restoration, and one school (7%) allowed UGs to place implants. In common with the Young *et al.* 1999 survey, the authors concluded that there was ongoing variation in teaching between UK institutions and that although improvements had been made, implant teaching was behind the level being provided in the US (further information regarding US teaching is described later in the article).

Blum *et al.* (2008)^11^ published a similar survey in the same year, but with clear differences in the reported UG implant teaching findings compared to the Addy *et al.* (2008) survey. The authors contacted the 13 UK dental schools which provided UG education (excluding Ireland and the two institutions which provided PG education only). All 13 participating UK dental schools responded that their UGs received implant teaching, with 100% providing lectures and covering the topic of osseointegration. Amongst other teaching opportunities, nine schools provided clinical teaching (69%), seven schools (54%) organised observation of restorative procedures, four schools (31%) organised restoration of implants, nine schools (69%) organised observation of implant surgery and four schools (30%) taught clinical maintenance.^10^ Only two schools (15%) provided simulation teaching. Aside from osseointegration, a variety of other implant topics were also reported to be taught (online Supplementary Table 1). One of the clear differences between the findings of this survey and the Addy *et al.* 2008 survey was the reporting by the responding institutions of 100% versus only 60% conducting lecture-based implant education. This discrepancy could be due to different compositions of institutions responding to each survey but, with no information on which institutions participated in which, this cannot be confirmed. Blum *et al.* (2008) also reviewed how many schools had implantology embedded in their UG curricula, therefore making implantology part of a structured plan for education (rather than optional or additional teaching). The significance of embedding implantology in a curriculum means that students are taught in a more structured way, clear learning outcomes regarding what students should know and be able to do by the end of an educational stage are met, and students are also assessed on their knowledge. 30% of schools responded that dental implantology was a formal part of their curriculum.

Both 2008 surveys identified a lack of consistency in the teaching at didactic, practical and clinical levels. Prioritising in an already overcrowded curriculum, staff or facilities requirements and resource constraints were suggested as possible reasons for the variation between institutions. To achieve further improvements in UK UG education, the authors concluded there was a need to remodel UG dental curricula to include implant dentistry.^11^

Chin *et al.* (2018) repeated the 2008 survey conducted by Addy *et al.* to establish whether there had been any change in the intervening decade in dental implant teaching in the UK and Ireland.^10^^,^^12^ 18 schools responded that implantology was taught in the UG curriculum, and an improvement in the range of teaching methods provided was also noted; 13 schools (81%) gave lectures, 14 schools (88%) provided simulation teaching and 13 schools (81%) provided some degree of clinical activity.^10^ The majority of clinical activity was centred on treatment planning (13 schools, 88%). Restorative procedures were observed in ten schools (63%) and performed in five schools (31%). Implant surgery was observed in 12 schools (75%) but only performed by UGs in one school (6%).

It was concluded that opportunities for clinical observation had improved whereas clinical hands-on experience remained low and similar to previous findings.

The most recent survey to examine and evaluate current UG dental implantology education focused on UK dental schools only (Hare *et al.* 2022).^13^ The response rate to this survey was lower than previous, with only 8/16 (50%) of schools engaging. Six of the eight schools (75%) had dental implantology embedded within their curricula. All eight responding schools (100%) provided lectures, and six schools (75%) provided pre-clinical simulation training. The clinical teaching reported in this survey was appreciably lower, with only one school (12%) providing observation of restorative procedures, one school (12%) allowing restoration of implants and two schools (25%) providing observation of implant surgery. These results may have been due to the lower response rate, or due to the survey being conducted during the coronavirus pandemic. The barriers to teaching were similar to those reported in previous surveys and included cost, limited time in the curriculum, lack of teaching staff expertise and training.

The importance of embedding dental implant education within UG curricula (as opposed to extra-curricular/optional teaching) was recognised in the consensus document following the British Society for the Study of Prosthetic Dentistry's 56^th^ Annual Conference 2009.^14^ The main conclusions were that:

- Implant therapy should be taught as a core item within treatment planning
- Foundation in basic implant theory and practice should be provided
- Clinical experience is fundamental and should be encouraged at undergraduate level.

Barriers were recognised including congested curricula, staff training, funding for support staff and patient selection. There have been some improvements in regard to embedding implants in undergraduate curricula in the UK. The Blum *et al.* survey (2008) reported only four of the 13 (30%) of responding schools had implantology embedded in curriculum, whereas the Hare *et al.* survey (2022) reported six of the eight (75%) responding schools had implantology embedded in curriculum.^10^^,^^12^ Even so, improvements on the 2022 findings are still required and a more comprehensive, consistent and detailed approach would be a beneficial ‘next step' to continue to guide and inspire UK dental schools.

### Current status of undergraduate education in the UK and Ireland in 2025

The reports following the aforementioned surveys suggests that there has been increased commitment to UG implant teaching in the UK over the past four decades, with an increase in the number of teaching hours, greater variety of teaching methods and more clinical experience opportunities. At the same time, it is clear that there are still considerable variations between the schools regarding the implant dentistry topics taught, the teaching methods employed for practical implant therapy and the UG exposure to these. The variation in survey responses and differing methodology between studies further makes it challenging to get a clear picture of the current UG implant teaching in the UK. It is not known from the most recent survey in 2022, for example, to which extent there has been an increase in the number of dental schools in the UK who routinely facilitate implant teaching and treatment provision for all undergraduates. In a globalised world, it would also be important to understand how UG dental education in the UK and Ireland compares to progress and status of dental schools internationally.

### Undergraduate education in Europe and beyond

Variation in UG implantology education is not unique to the UK and Ireland and it has also been a topic of discussion at a European level for the past two decades. This has been largely led by the Association for Dental Education in Europe (ADEE) who organised the first and second European workshops to review implant education, which resulted in the publication of UG curriculum consensus guidelines (Mattheos *et al.* 2009, Mattheos *et al.* 2014), two European surveys (De Bruyn *et al.* 2008 and Koole *et al.* 2014) exploring the status of dental implantology education in Europe, and a systematic review to assess UG implant education globally (Koole and De Bruyn 2014).^15^^,^^16^^,^^17^^,^^18^^,^^19^

In 2008 the first European Workshop on Implant Dentistry University Education was held in Prague to promote a consensus on guidelines for UG in dental implantology aimed at improving and standardising implant education.^19^ One of the Workshop groups focused on the teaching and assessment of implant dentistry at both undergraduate and PG levels. For undergraduate education, a required knowledge base with a list of ten objectives and outcomes upon graduation were agreed (Table 1). The outcomes broadly stated that graduates should have knowledge regarding implant treatment planning, stabilisation, tissue integration, treatment phases, tissue augmentation, restoration, risks, complications and maintenance. Suggestions for assessment to test this knowledge included reflective assessment, written and oral examination. Specific assessments related to the different objective and outcomes, however, were not discussed. Furthermore, no recommendations were included regarding the type of educational activity (e.g., lectures, simulation or clinical practice). Another workshop group expanded on the suggested knowledge that should be included in UG education.^20^ The published guidance described in detail the topics within an UG curriculum (Table 1) as well as the associated, recommended content and format designed for educators to implement easily. Although the breadth of recommended topics was extensive, the suggested content was designed to be preparatory to develop foundational knowledge. These guidelines provided comprehensive detail for educators to help tailor local UG curricula. As already described, however, the uptake in the UK of the recommendations has been variable. This might be due to the previously mentioned barriers (e.g., staff expertise). It is also possible that the breadth and depth of content was too much for some/most UK institutions to implement.

**Table 1:** Required knowledge base for undergraduate implant dentistry from the 1^st^ European Consensus Workshop on Implant Dentistry University Education: Prague, June 2008^19^^,^^20^

**First European consensus workshop on implant dentistry university education for undergraduate students**		
Objectives and outcomes sought (Mattheos *et al*. 2009)^19^	Theoretical knowledge (Hicklin *et al*. 2009)^20^	
Be capable of deciding on the most appropriate treatment option to replace missing teeth whether that should be a removable or FDP supported by teeth or an implant supported reconstruction, always with emphasis on evidence-based treatment. Have knowledge and understanding of how to establish a clinically healthy oral environment through plaque control, caries removal and treatment of periodontal and other oral pathologies of patients who are going to receive oral implants. Be aware of the biomechanical interactions between the tissues and the implant material. The interface between the living tissues and the dental implant is critical for many aspects of successful implant therapy. Have adequate knowledge and understanding of the surgical and prosthetic procedures involved in implant treatment is mandatory, as well as anticipating possible complications. Successful dental implants necessitate careful treatment planning including differentiation between low, medium and high-risk situations. Be aware of related treatment options such as guided bone regeneration and bone grafting including the indications and contraindications for such procedures. Have adequate knowledge and understanding of removable as well as ﬁxed prosthodontics, as awareness of biomechanical aspects of implant borne reconstructions is critical for their long-term success. Have knowledge and understanding of the possibilities and limitations with respect to the aesthetic outcome of implant treatment. Be aware of the risk of, as well as treatment options for, peri-implant tissue destruction because of a combination of infection and inﬂammation. Be aware of the risk of, as well as treatment options for, different prosthodontic problems leading to peri-implant tissue destruction. Have knowledge and understanding of the criteria for success of oral implants as well as of the long-term prognosis of osseointegrated implants and associated restorations. This includes the ability to diagnose and manage failing and failed implants and associated restorations.	**1) Implant treatment overview** Treatment strategy Treatment planning Medical history Treatment of oral diseases Surgical phase Intermediate phase Reconstructive phase Maintenance Indications Principles of implant placement **2) Osseointegration** Histology Chemical characteristics of implant materials Macroscopic design Surface structure Implant surface modiﬁcation **3) Soft tissue interface** Anatomy of peri-implant tissues Conditions for a functional peri-implant mucosa Clinical aspects Maintenance **4) Clinical and diagnostic information** **5) Aesthetic considerations** **6) FDP on teeth vs implant** **7) Materials for dental implants** Titanium Zirconia **8) Surgical procedures** Pre-medication and anaesthesia Incision and ﬂap design Implant positioning Implant bed Wound healing Follow-up	**9) Surgical complications** Implant-speciﬁc complications Bleeding and haematoma Infection Damage to neighbouring teeth Damage to nerves Liability **10) Management of bone and soft tissues for implant site development** Bone grafting Distraction osteogenesis Soft tissue management **11) Prosthetic aspects: removable** Edentulous lower jaw Edentulous upper jaw Complications Partially edentulous **12) Prosthetic aspects: ﬁxed** Cemented Screw-retained **13) Biomechanical aspects** Tactile sensitivity with implants Tooth-implant borne reconstructions Impact of bruxism Necessary number and length of implants **14) Aetiology, pathogenesis, prevention and therapy of peri-implantitis** Definitions Microbial colonisation Peri-implant mucositis Peri-implantitis Diagnostic aspects Treatment of peri-implant mucositis and peri-implantitis Possible reasons for bone loss other than peri-implantitis **15) Long-term results of implant-supported reconstructions** Survival and success Maintenance in the presence of biological complications Removal of the implant

Following the first European workshop on implant dentistry in 2008, a European-wide survey was conducted to identify the status of UG and PG implant dentistry education, the results of which are summarised in online Supplementary Table 1.^14^ Here, 73 participants from an ADEE workshop (Prague, 2008) were surveyed, with a response rate of 67%. The responses represented 35 institutions in 18 European countries. While all respondents reported teaching UG dental implantology, there was variability between institutions. 100% of responders provided theoretical teaching, 66% pre-clinical (simulation) teaching, 53% had clinical experience assisting surgery, 44% had clinical experience assisting implant prosthetics and 28% completed implant work on their own patients. It was not clear from this study how many schools were following the 2008 European consensus guidelines in relation to the learning outcomes defined. In preparation for the second ADEE workshop in implant dentistry education, a subsequent similar survey by Koole *et al.* (2014) (online Supplementary Table 1) was conducted to review UG and PG implant dentistry education in Europe five years after the ADEE consensus guidelines were published.^17^ Here, 105 participants from an ADEE workshop were surveyed, with a 50% response rate. The responses represented 44 institutions from 20 different countries, 43 of which provided UG education, all of which provided UG implant education. A slight decrease (93% compared to 100%) was reported for theoretical based implant education, whereas increases in pre-clinical (77% compared to 65%) and clinical education were observed. 73% provided clinical experience assisting surgery, 66% offered clinical experience assisting implant prosthetics and 52% completed implant work on their own patients (compared to 28% in 2009). The number of reported hours spent on implantrelated education was also higher. Respondents reported an average of 74 h (range 4–288 h) total time in the undergraduate curriculum spent on implant dentistry related topics. This suggested a marked increase compared to 36 h (3–120 h) in 2009. Again, it was not reported how many schools were following the 2008 European consensus.

In comparison to these 74 average hours in other areas of Europe, the five UK schools provided ten hours and one UK school providing 11–20 hours of UG implant teaching are clearly still at the lower end.

With the aim of gaining a better and more detailed understanding of competences and barriers within UG implant education globally, Koole and De Bruyn (2014) performed a systematic review of publications between 2008 and 2013.^15^ The 37 studies included representative data from studies conducted within the continent of Europe (including Denmark, France, Germany, Switzerland) as well as Canada, India, Indonesia, Ireland, Japan, UK, and USA. One hundred percent of programmes included the scientiﬁc basis for implant dentistry in their curriculum, but there was otherwise no consistency between programmes. While basic theoretical education was by far the most common, pre-clinical exercises were less frequent, and clinical education via assisting or self-performed implant-related treatment was only offered by a limited number of programmes. Table 2 details the array of topics taught as well as methods of different educational activities. The authors concluded that although implant dentistry was increasingly integrated in undergraduate curricula, it was difficult to establish to what extent each aspect of implant dentistry was taught, particularly the specifics regarding pre-clinical and clinical activity. Furthermore, as the reporting in many of the studies was vague and lacked detail on programme content, the results gathered in the systematic review cannot be considered complete. Universities at large showed difﬁculties in fully integrating implant dentistry within their UG programmes where barriers again included already overcrowded curriculums and not being considered a priority, clinical aspects too complex to teach, insufficient financial resources and training of staff for teaching and supervision.

**Table 2:** Didactic UG implant education teaching methodologies taken from Koole and De Bruyn 2014^15^

**All listed in order from most to least frequent**			
**Topics** **(no. studies reported)**	**Teaching activities (no. studies reported)**		
	**Theoretical**	**Preclinical**	**Clinical**
Treatment planning (5) Survival and success rates (3) General introduction (3) Indications and contraindications (3) Bone grafting (3) Tissue integration (2) Biomaterials (2) Maintenance (2) Restoration (2) Treatment of failing implants (2) Surgical aspects (2) Pre-surgical assessment (2) Sinus grafting (2) Post-surgical care (2) CBCT planning (2) Implant types (1) Surface topography (1) Patient information (1) Soft tissue management (1) Prosthetic complications (1) Surgical techniques (1) Navigated surgery (1) Suturing (1) Immediate loading (1) Complications (1)	E-learning (5) Selected literature (4) Self-directed learning (2) Lectures (2) Problem-based learning (2) Tutorial-based learning (1) Student–teacher-centred education (1) Videos (1) Internet-based information (1) Text-books (1)	Training on simulated models (2) Implant planning (1) Implant osteotomy preparation on pig heads (1) Surgical drills (1)	Restoration of implants (4) Observation of implant surgery (4) Chairside assisting implant surgery (3) Clinical examination for diagnosis and treatment plan (3) Implant placement (2) Assisting in restorative procedures (2) History taking (2) Pre-surgical care (1) Participate in live demonstration by the instructor (1) Extractions (1)

Comparing UG implant education in the UK directly with other countries around the world is challenging due to lack of consistent data. A 2014 survey from the USA did, however, include data in its comparisons which can be compared with that in the UK.^21^ Ninety percent of the responding US institutions provided simulation exercises (the UK was at 75–80%), 96% of US institutions provided clinical teaching to restore dental implants (compared to 12–31% in the UK), and 94.2% of US institutions reported their programmes included direct patient care under supervision (in contrast to UK at 31% or 88% if including treatment planning).^8^^,^^10^^,^^11^^,^^13^ Although there are significant differences between university education in the UK and the USA (such as university admissions and educational funding) these data shows the ambitious outcomes for UG implant education achieved elsewhere in the world. Unfortunately, similar data are not accessible to compare UK UG implantology education with other countries.

While it is not possible to directly compare UG dental implant education in the UK to education elsewhere in the world, it does appear that the UK is falling behind in respect of teaching hours, variety of teaching methods and provision of clinical experience. It is important to be cautious when making such comparisons, of course as unique local, national educational and operational pressures have not been accounted for. It seems clear, nevertheless, that further momentum and evolution in this area is required to encourage the necessary progression of UK UG implant education,

### The impact of dental implant education at undergraduate level

It is evident that finding space for UG implant education in already crowded curricula is a challenge. There is evidence, however, that UG education in dental implantology has beneficial effects for both patients and the wider profession.

Pre-clinical and clinical implant experience offered at an UG level has shown to positively inﬂuence dentists' involvement in implant therapy after graduation. One US study showed that students who had implant education included in their UG level training were more inclined to offer implants, restore implants and go on to place implants compared to dentists that did not receive the same implant education within the same dental school.^22^ Another US study compared students who received implant training as part of their UG level education with those in different schools that did not.^23^ The students who received implant education performed two times more implant restorations, approximately four times more implant placements and made more frequent implant referrals to surgical specialties once they were practicing dentists. Furthermore, the likelihood of taking on PG implant education was greater in the cohort who received UG level training.

Although there are no published studies from the UK evaluating the influence of UG education on subsequent practice, a recent unpublished PhD thesis, investigating current and future challenges for dental implant practice and education in the UK, concluded that dentists provided maintenance 9.2 times more if they learnt to place and/or restore implants at an UG level.^24^ This research evaluated the opinion of 87 dentists with around 5–21 years' experience using qualitative surveys. The perception of the majority of the dentists who participated in the survey was that implant education was still inconsistent in the UK. As a result, the participants felt this did not instil confidence amongst UK dentists to manage dental implant patients. An older survey from 2006, investigating the opinion and experience of 106 UK GDPs with regards to dental implantology education, established that the majority (75%) had not been taught oral implantology as undergraduates.^25^ Although this is unsurprising considering the time period in which the GDPs completed their UG education, the consequences of not having appropriate dental implant education were acknowledged. These included little to no confidence as to whether implant treatment was appropriate in any given case (37.5% of respondents), not including implants as a legitimate treatment option, making inappropriate referrals and not undertaking implant maintenance with only 66% feeling they could provide this. These consequences were in direct conflict with 98% of the responding GDPs believing that they should have a role in discussing the option of implants to patients where appropriate.

There is very little research into the perceived readiness of UK dental students for managing dental implants in general dental practice. One unpublished study by Harris (2012) surveyed final year dental UG students in the Bristol and Peninsula Dental Schools regarding implant education.^26^ The majority of UG students reported they were only moderately comfortable discussing implants as a treatment option, conducting patient assessment for dental implants and maintaining dental implants (including oral hygiene and debridement) and no students reported they were very comfortable. Lower levels of clinical confidence are well recognised in final year dental students, particularly for complex procedures that are least practised. As provision of dental implants is considered a more ‘advanced' area of dentistry, it could be argued that the moderate confidence levels reported by Harris 2012 should be expected.^16^ Nevertheless, procedures that are ‘simple' and performed regularly during UG education have been shown to result in improved confidence.^16^ Equally, it could therefore be argued that regular exposure to the straightforward aspects of dental implantology (such as discussing them as a treatment option and undertaking routine implant maintenance) would increase confidence upon graduation, and thus enable graduates to fulfil the roles and responsibilities expected of a GDP.

As clinical implant dentistry continues to evolve, so too does UG dental education. Much of the consideration over the past few decades has been about what to teach (through competencies and learning outcomes), while more recently the question of how to teach has come more into focus. It has been shown that simply defining the expected learning outcomes for skills courses is not enough.^27^ There is a need to continually consider the active learning processes required to accomplish certain learning outcomes, and to understand that a range of learning styles (e.g., self-directed, practical, problem-based and reflective learning) are often required to develop theoretical, practical and clinical skills. Furthermore, deconstructing learning outcomes into tasks which are reinforced longitudinally over a programme has been suggested as a more effective way to develop curious, independent learners.^28^ It is therefore important to not just consider the appropriate content of UK UG education but also ensure that guidance for educators continues to evolve and include more modern approaches to dental UG education.

## Conclusions

Planning and delivering relevant and effective UG education is a constant challenge for educational institutions. Developing a workforce of dental professionals with the appropriate skills to effectively care for the needs of the wider population requires continued reflection on educational strategy and guidance. With the ever-changing landscape within dentistry and dental implantology, it is important that UG education evolves at a similar pace so that graduating dentists are trained to provide effective care for patients with dental implants. Efforts to minimise discrepancies in implant education at an UG level are necessary to prevent inconsistencies in dental care, both nationally and internationally. The work that professional societies and organisations have contributed to educational guidance for implant education is imperative, and continued reflection and collaboration is needed to continue this important role.

Previous guidelines have been instrumental in progressing the standard of international, national and local education, and this has been demonstrated by numerous surveys over the last four decades. However, variations in survey design and reporting makes it difficult to directly compare outcomes and quantify improvement. Standardised methodology and reporting based on consensus guidelines would help consistency and comparison for future studies.

Despite notable progress in UG dental implant education over the last 40 years, the UK still falls behind other countries in terms of hours and breadth of teaching provided. As the UK moves from the GDC *Preparing for Practice* to the *Safe Practitioner Framework,* it is a timely opportunity to reflect on UG implant education and establish how further improvements can be made. Continued guidance on curriculum content is essential, and guidance on learning styles, format, environment and effective assessment are also paramount to encouraging schools to make effective curriculum changes.

## Supplementary Information

Summary of the results of surveys conducted to review dental implant UG education in the UK and Europe Row 1-6 relate to UK surveys. Rows 7 and 8 presents the summary from two recent European surveys. (DOCX 24KB)
