# Supplementary material for: Undergraduate dental implantology education in the United Kingdom – looking to the past to plan for the future
Source: Br Dent J. 2026 May 8;240(9):606–12. doi: 10.1038/s41415-026-9627-5 (PMC13156026; doi:10.1038/s41415-026-9627-5)
Supplement: Supplementary file 1 — Summary of the results of surveys conducted to review dental implant UG education in the UK and Europe Row 1-6 relate to UK surveys. Rows 7 and 8 presents the summary from two recent European surveys. (DOCX 24KB) [file 41415_2026_9627_MOESM1_ESM.docx]

| Author / year | Dental Schools included | Response rate % (schools) | Implant dentistry taught  % (schools) | Theoretical (lecture-based)  % (schools) | Pre-clinical (simulation)  % (schools) | Clinical teaching % (schools) | | | | Topics taught  (highest to lowest frequency) | Didactic teaching time | Challenges |
| --- | --- | --- | --- | --- | --- | --- | --- | --- | --- | --- | --- | --- |
|  |  |  |  |  |  | Treatment planning | Restorative | Surgical | Maintenance |  |  |  |
| Watson  1993^8^ | 16 UK  2 Ireland | 94 (17/18) | 70 (12) | 70 (12) | Not specified | 23 (4) | 35 (6) | 35 (6) | Not specified | Not specified | 1-5 lectures |  |
| Young et al.  1999^9^ | 16; no additional information given | 100 (16/16) | 100 (16) | 100 (16) | Not specified | Observation (activity not specified) 25 (4) | | | | Not specified | Not specified | Not specified |
| Addy et. al  2008^10^ | 15; no additional information given | 100 (15/15) | 87 (13) | 60 (9) | 53 (8) | 46 (7) | Observation 46 (7)  Provision 27 (4) | Observation 33 (5)  Placement 7 (1) | Not specified | Not specified | • 47% (7) 4-6 sessions  • 20% (3) 1-3 sessions  • 20% (3) > 6 sessions | • Funding  • Lack of time  • Staff expertise |
| Blum et al  2008^11^ | 13 UK – UG teaching only | 100 (13/13) | 100 (13) | 100 (13) | 15 (2) | Not specified | Observation 54 (7) Provision 31 (4) | Observation 69 (9) | 30 (4) | Osseointegration 100% (13)  Implant surgery 100% (12)  Dental pre-surgical assessment 92% (12)  Radiographic/image evaluation 92% (12)  History of implants 84% (11)  Implant site selection 84% (11)  Classiﬁcation and types of dental implants 84% (11)  Implant surface treatment 84% (11)  Biomechanics/biomaterials 77% (10)  Treatment planning 77% (10)  Medical pre-surgical assessment 77% (10)  Surgical complications and management 69% (9)  Occlusion 62% (8)  Post-surgical care 62% (8)  Craniofacial application of implants 54% (7)  Prosthetic complications and management 46% (6)  Current research and developments 46% (6)  S Failing implants 46% (6)  crew vs. cemented restoration 38% (5)  Patient education 31% (4)  Immediate implant loading 31% (4)  Other topics 8% (1) | • 85% (11) 10 hours  • 15% (1) 11-20 hours | Not specified |
| Chin et al.  2018^12^ | 16 UK  2 Ireland | 88% (16/18) | 100 (16) | 81 (13) | 88 (14) | 81 (13) | Observation 63 (10)  Provision 31 (5) | Observation 75 (12)  Placement 6 (1) | 6 (1) | Not specified | • 56% (9) 4-6 sessions  • 31% (5) 1-3 sessions  • 13% (2) > 6 sessions | • Lack of time  • Staff expertise |
| Hare et al.  2022^13^ | 16 UK – UG teaching only | 50% (8/16) | 100 (8) | 100 (8) | 75 (6) | Not specified | Observation  12 (1)  Provision 12 (1) | Observation 25 (2) | Not specified | Failing implants 87% (7)  Prosthetic complications and management 87% (7)  Implant patient education 75% (6)  Screw vs. cemented restoration 75% (6)  Surgical complications and management 75% (6)  Current research and developments 62% (5)  Post-surgical care 50% (4)  Occlusion 50% (4)  Immediate implant loading 37% (3)  Craniofacial application of implants 37% (3) | Not specified | • Funding  • Lack of time  • Staff expertise |
| De Bruyn et al. 2009^15^ | 73 participants representing 34 institutions in 18 countries, 43 with had UG teaching | 67 (49/73) | 100 (43) | 100 (43) | 65 (28) | Not specified | Observation  44 (19)  Provision  35 (15) | Observation 51 (22)  Placement  5 (2) | Not specified | Not specified | 36 h (range 3 to 120 h) | - Lack of time  - Cost  - Staff shortage |
| Koole et al. 2014^17^ | 105 participants representing 46 institutions in 20 countries, 44 with UG teaching | 50 (52/105) | 98 (43/44) | 93 (41) | 77 (34) | Not specified | Observation  66 (29)  Provision  52 (23) | Observation 73 (32)  Placement  25 (11) | Not specified | Not specified | 74 h (range 4 to 288 h) | - Lack of time  - Cost / funding  - Limited patients  - Limited staff  - Government regulations  - Liability / insurance |

Table 1 – Summary of the results of surveys conducted to review dental implant UG education in the UK and Europe Row 1-6 relate to UK surveys. Rows 7 and 8 presents the summary from two recent European surveys.
